# Laboratory Modeling of SARS-CoV-2 Exposure Reduction Through Physically Distanced Seating in Aircraft Cabins Using Bacteriophage Aerosol — November 2020

**DOI:** 10.15585/mmwr.mm7016e1

**Published:** 2021-04-23

**Authors:** Watts L. Dietrich, James S. Bennett, Byron W. Jones, Mohammad H. Hosni

**Affiliations:** ^1^Division of Field Studies and Engineering, National Institute for Occupational Safety and Health, CDC; ^2^Department of Mechanical and Nuclear Engineering, Kansas State University, Manhattan, Kansas.

Aircraft can hold large numbers of persons in close proximity for long periods, which can increase the risk for transmission of infectious disease.* Current CDC guidelines recommend against travel for persons who have not been vaccinated against COVID-19, and a January 2021 CDC order requires masking for all persons while on airplanes.^†^^,^^§^ Research suggests that seating proximity on aircraft is associated with increased risk for infection with SARS-CoV-2, the virus that causes COVID-19 (*1*,*2*). However, studies quantifying the benefit of specific distancing strategies to prevent transmission, such as keeping aircraft cabin middle seats vacant, are limited. Using bacteriophage MS2 virus as a surrogate for airborne SARS-CoV-2, CDC and Kansas State University (KSU) modeled the relationship between SARS-CoV-2 exposure and aircraft seating proximity, including full occupancy and vacant middle seat occupancy scenarios. Compared with exposures in full occupancy scenarios, relative exposure in vacant middle seat scenarios was reduced by 23% to 57% depending upon the modeling approach. A 23% exposure reduction was observed for a single passenger who was in the same row and two seats away from the SARS-COV-2 source, rather than in an adjacent middle seat. When quantifying exposure reduction to a full 120-passenger cabin rather than to a single person, exposure reductions ranging from 35.0% to 39.4% were predicted. A 57% exposure reduction was observed under the vacant middle seat condition in a scenario involving a three-row section that contained a mix of SARS-CoV-2 sources and other passengers. Based on this laboratory model, a vacant middle seat reduces risk for exposure to SARS-CoV-2 from nearby passengers. These data suggest that increasing physical distance between passengers and lowering passenger density could help reduce potential COVID-19 exposures during air travel. Physical distancing of airplane passengers, including through policies such as middle seat vacancy, could provide additional reductions in SARS-CoV-2 exposure risk.

The study consisted of three components. The first involved analysis of data on virus aerosol dispersion in aircraft cabin mock-ups from a previous study conducted at KSU during July–August 2017 as part of a pandemic influenza research initiative (*3*). Next, these data were used to create a regression model to estimate the reduction in aerosol concentration as distance from a source increased. Finally, these regression models were applied to conceptual aircraft seating scenarios to simulate the reduction in exposure resulting from vacant middle seats in an aircraft cabin. Laboratory experiments were performed with bacteriophage MS2 virus obtained from the American Type Culture Collection.^¶^ Bacteriophage MS2 has frequently been used as a surrogate for pathogenic viruses in aerosolization studies (*4*) and was used to approximate the airborne dispersion of SARS-CoV-2. During the aerosol dispersion study at KSU, mannequins with realistic passenger heat emission were seated in the cabin mock-ups, and then MS2 aerosol was introduced from a source location and collected at six different sample locations in the cabin. This process was repeated four times: twice in a single-aisle cabin and twice in a twin-aisle cabin (Figure 1), resulting in 24 total samples.** Because these data were collected before the COVID-19 pandemic, the effects of passengers wearing masks on the aerosol dispersion behavior were not measured. These viral aerosol data were then used to create a nonlinear regression model^††^ which assesses the association between the number of plaque-forming units (PFUs) (evidence of the presence of viable virus) and the distance between source and sample locations. For both single-aisle and twin-aisle scenarios, findings from the nonlinear regression model indicate that the number of PFUs declined exponentially with increasing distance (Supplementary Figure, https://stacks.cdc.gov/view/cdc/104935).

**FIGURE 1:**
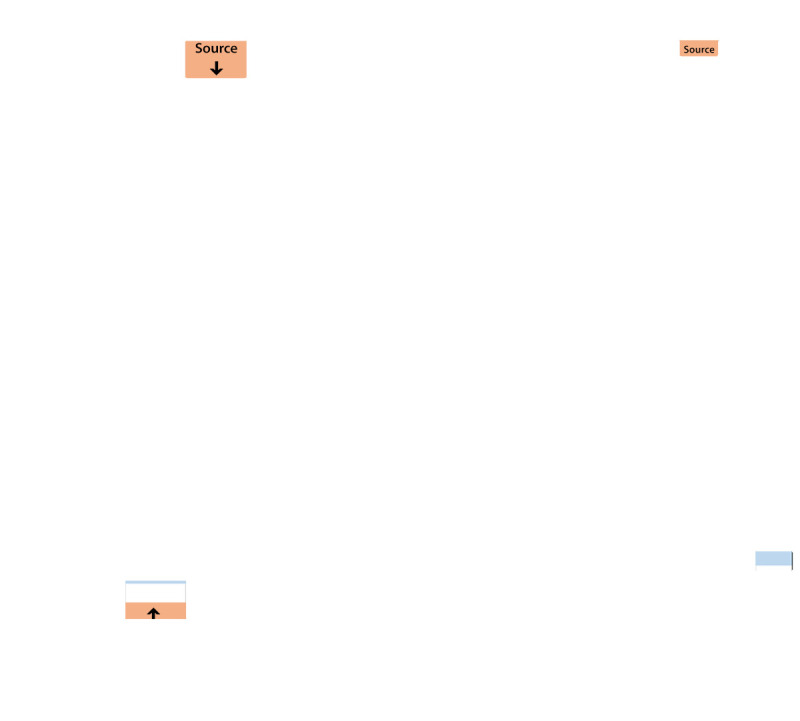
Diagram of aircraft cabin configurations and source and sampling locations to assess exposure to aerosolized bacteriophage MS2 virus as a surrogate for airborne SARS-CoV-2 exposure in single-aisle and twin-aisle cabins* — Kansas State University, July–August 2017^†^ **Source:** Modified with permission from Lynch JA, Bennett JS, Jones B, Hosni MH. Viral particle dispersion and viability in commercial aircraft cabins. In: 2018 American Society of Heating, Refrigerating and Air-Conditioning Engineers Annual Conference Proceedings; June 23–27, 2018; Houston, TX; 2018. **Abbreviations:** aft = back of the plane; fore = front of the plane; source = aerosol source; X = sampling location. * For both single-aisle and twin-aisle cabin scenarios, two different source configurations were assessed for placement of infectious passengers: one with the source at the front of the plane in an aisle and one with the source in a seat. Each configuration consisted of six total sampling locations, for a total of 24 samples. ^†^ Data were collected at Kansas State University during July–August 2017 as part of a pandemic influenza research initiative.

In November 2020, CDC applied this data-driven model to simulate the protective effect of a vacant middle seat versus full aircraft occupancy. Two analytical approaches were used. Both approaches analyzed reductions in relative exposures (the number of PFUs divided by the maximum predicted value) rather than absolute exposure.

The first approach considered only the extra distance between passengers created by the vacant middle seat. The regression model estimated exposure as a function of distance to assess the exposure reduction of moving an adjacent passenger one seat further away from an infectious passenger, leaving an empty middle seat between them. The distance effect was explored further to simulate the total exposure reduction for groups of passengers up to and including a full simulated cabin of 120 seats.^§§^ A total of 300 simulations were tested using Monte Carlo methods, where the number (one to three) and placement of infectious passengers were varied. The total exposure reduction for all passengers in the cabin was predicted by placing a source at an arbitrary seat location and applying the regression model to calculate relative exposure at all other seat locations, which were summed to obtain a total exposure for the cabin.

The second approach combined the distance effect predicted by the regression model and the reduced occupancy effect predicted by simple probability estimation, as these are inseparable in realistic arrangements of infectious passengers and other passengers. When simply defining exposure risk as reduced occupancy, a vacant middle seat reduced exposure by an estimated 33% compared with full occupancy, in single-aisle, three-seats-per-side cabins, because there are 33% fewer potentially infectious passengers.

The first approach predicted a 23% exposure reduction by moving an adjacent passenger one seat further away from an infectious passenger. The total reduction in relative exposure for a full 120-seat cabin yielded reduction of 35.0%–36.4%, 35.1%–38.2%, and 35.9%–39.4% for one, two, and three infectious passengers, respectively, depending on their seating pattern. All sources were placed in window or aisle seats such that the potential reduction in number of infectious passengers onboard from vacant middle seating was not considered (Figure 2). The second approach was applied to a cluster of nine infectious passengers (including three in middle seats) among 18 total passengers in three rows (Figure 3). When the infectious and other passengers who would have had middle seats were removed, leaving six infectious passengers out of 12 total passengers remaining in the window and aisle seats, a 57% exposure reduction was observed.

**FIGURE 2:**
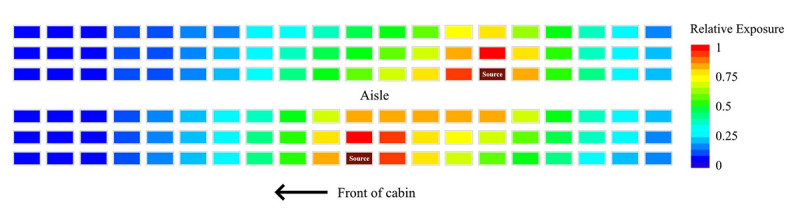
Estimated reduction in relative exposure to aerosolized bacteriophage MS2 as a surrogate for SARS-CoV-2 through physically distanced seating in a single-aisle, 20-row simulated aircraft cabin* — November 2020 * A total of 300 simulations in which the number (one, two, or three) and placement of infectious passengers varied were tested using Monte Carlo methods. The simulated cabin had a single aisle, with 20 rows and three seats per side (120 total seats); the distance between rows was 3 ft (0.9 m); the distance between adjacent seats was 1.6 ft (0.5 m). In the source configuration shown here, the total reduction in exposure with vacant middle seats was calculated to be 35.4%.

**FIGURE 3:**
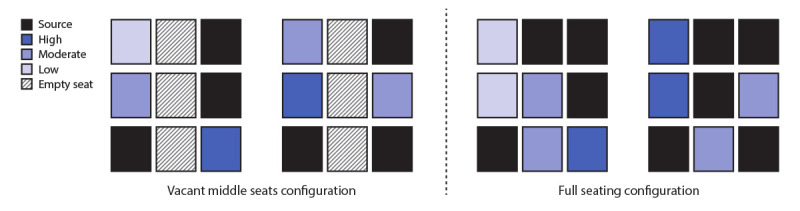
Relative estimated reduction in exposure to bacteriophage MS2 as a surrogate for SARS-CoV-2 through physically distanced seating in a single-aisle, three-row section of an aircraft cabin with full occupancy* compared with vacant middle seats^†^ — November 2020 * A cluster of nine infectious passengers (including three in middle seats) among 18 total passengers in three rows. Removing the infectious and other passengers who would have had middle seats, leaving six infectious passengers out of 12 total passengers remaining in the window and aisle seats, resulted in an estimated 57% reduction in SARS-CoV-2 exposure. Exposures were as follows: 21.1 for six noninfectious passengers in the configuration with no middle seats; 48.7 for the nine noninfectious passengers with full occupancy. ^†^ The local prevalence (the percentage of passengers in the three rows who are infectious) was held constant in the comparison to reasonably account for the fact that keeping middle seats vacant is expected to prevent both infectious and noninfectious passengers from sitting there.

## Discussion

This laboratory-based model predicts a 23% to 57% reduction in exposure to viable virus particles when middle seats on an airline are kept vacant. This range is comparable to that reported in another study that used computational fluid dynamics simulation and considered cabin ventilation rates per passenger to show that keeping middle seats vacant reduced the risk for airborne infection by 45%.^¶¶^ Studies of tracer gas/particle dispersion generally indicate that distance is an important determinant of contaminant exposure on aircraft (*5*,*6*), including showing that airborne concentration decay with distance is similar for various contaminant types and closely mirrors infection patterns on aircraft; this finding supports the use of bacteriophage MS2 as a surrogate for SARS-CoV-2 exposure.*** Further, a recent investigation of SARS-CoV-2 transmission on an international flight found that seating proximity was strongly associated with infection risk: 75% of infected passengers were seated within two rows of the symptomatic passenger who likely originated the outbreak (*1*).

Aircraft cabin environmental control systems (ventilation systems) are designed to deliver amounts of clean air per occupant that conform to various standards.^†††^ When these standards are adhered to, most virus particles are removed within several seat rows from a source on an aircraft, and the recirculated portion of the air supplied to each passenger has passed through high efficiency particle air (HEPA) filters.^§§§^ As aircraft ventilation removes airborne contaminants, it also causes some turbulent dispersion. This spreading effect of aerosols is larger than transient flows created by passenger or crew movement in the aisles under typical cruise conditions (*7*). Physical distancing is difficult on crowded flights, and sitting within 6 ft of others, sometimes for hours, might increase risk for SARS-CoV-2 exposure. To reduce this risk, the CDC order issued in January 2021 requires the wearing of masks by travelers to prevent spread of COVID-19, including all passengers on aircraft traveling into, within, or out of the United States, and recommends against travel for all unvaccinated persons.

It is important to recognize that the current study addresses only exposure and not transmission.^¶¶¶^ The impact of masking also was not considered in the current aerosol analysis because masks are more effective at reducing fomite and droplet exposures than aerosol exposures (*8*,*9*). A case study of COVID-19 transmission on a flight with mandated mask wearing (*10*) suggests that some virus aerosol is emitted from an infectious masked passenger, such that distancing could still be useful. The findings in these studies indicate that masking seems to not eliminate all airborne exposures to infectious droplets and aerosols and support the importance of multicomponent prevention strategies as good practices; combining the effects of masking and distancing is more protective than either by itself.

The findings in this report are subject to at least four limitations. First, data were collected under higher relative humidity conditions in the laboratory than would be present during flight. Droplet evaporation into aerosol is more rapid under lower relative humidity. Because aerosols travel farther than droplets, the current study might underpredict the aerosol spread in an actual cabin environment. The slower evaporation in the current study might then overpredict the observed effect of distancing because this more rapid decrease makes estimated distance effects larger. Second, in the data used to build the regression models, most of the variability was within approximately 5 ft of the infection source. Although this near-zone variability weakens the quantification of the effect of short distances, the equations were the statistical best fit and had coefficient of determination (R^2^) values (the percentage of the response variable variation explained by a model) above 70%, suggesting that the distance model explained most of the observed virus concentration behavior. Third, the use of spray bottles to emit droplets, followed by 5 minutes of air sampling, might not fully represent the variety of respiratory events that could transmit virus (e.g., exhalation, talking, coughing, and sneezing). Mandated mask use further alters the human respiratory source, making the relative exposure approach used here an important way to diminish bias related to release volume. Finally, the study only assessed aerosols, not fomites and droplets. Exposures decrease more rapidly with distance for these exposure paths; therefore, distancing would have an even larger protective effect than that observed in this study.

Based on a data-driven model, approaches to physical distancing, including keeping middle seats vacant, could reduce exposure to SARS-CoV-2 on aircraft. The extent to which exposure reduction might decrease transmission risk is not yet understood. Current CDC guidelines recommend against travel for persons who have not been vaccinated and require masking for all persons while on aircraft. Physical distancing of aircraft passengers, including through policies such as middle seat vacancy, could provide additional reductions in SARS-COV-2 exposure risk. This study could help inform future modeling of transmission risk, which might encompass determinants that were not fully explored here such as mask use, virus characteristics, and host characteristics, such as vaccination status.

**Summary**

**What is already known about this topic?**

Aircraft can hold large numbers of persons in close proximity for long periods, which are conditions that can increase the risk for transmitting infectious diseases.

**What is added by this report?**

Based on laboratory modeling of exposure to SARS-CoV-2 on single-aisle and twin-aisle aircraft, exposures in scenarios in which the middle seat was vacant were reduced by 23% to 57%, compared with full aircraft occupancy, depending upon the model.

**What are the implications for public health practice?**

Physical distancing of airplane passengers, including through policies such as middle seat vacancy, could provide additional reductions in risk for exposure to SARS-CoV-2 on aircraft.

## Footnotes

*
https://www.who.int/whr/2007/en/

†
https://www.cdc.gov/coronavirus/2019-ncov/travelers/travel-during-covid19.html

§
https://www.cdc.gov/quarantine/pdf/Mask-Order-CDC_GMTF_01-29-21-p.pdf

¶
https://www.atcc.org

** The laboratory cabin environments were a five-row section of an actual Boeing 737 fuselage and an 11-row section of a geometrically accurate mock-up, including original equipment seats, of a Boeing 767 cabin. Ventilation systems used actual Boeing 737 (single aisle) and 767 (twin aisle) equipment where practical, but with no jet engine, so that supply ventilation came from conditioned air in the laboratory rather than from a jet engine. Bacteriophage MS2 virus aerosol was introduced by spray bottle from a source location using three closely timed, consecutive sprays, totaling approximately 3.7 mL. For both single-aisle and twin-aisle cabin scenarios, two different source configurations were assessed: one with the source at the front of the plane and one with the source at the back. Each configuration consisted of six total seat locations; a total of 24 samples were evaluated. Viral aerosol samples were collected in bioaerosol liquid (10% glycerol in 1× phosphate buffered saline) impingers. These devices collected aerosol by drawing air though a small volume of liquid for 5 minutes; samplers were held in place with clamp and stand assemblies on seats, with the impinger intake facing forward to approximate seated head-height for an average adult. Collection fluid aliquots were evaluated for viral particle presence by plaque assay, and the number of plaque-forming units (PFUs) was considered proportional to the airborne concentration references of viable virus.

†† The virion data were fitted to exponential regression equations of the form, C = Ae^BD^, with number of PFUs (C) as the dependent variable and the distance between spray and sampling locations (D) as the independent variable. A and B are constants determined by regression analysis, as was the coefficient of determination, R^2^.

§§ This simulated cabin was single-aisle, with 20 rows and three seats per side, for a total of 120 seats; the distance between rows was 3 ft (0.9 m); the distance between adjacent seat centers was 1.6 ft (0.5 m). This number of rows is on the lower end of a typical Boeing 737 coach cabin, depending on the specific design. The total exposure reduction for all passengers in the cabin was predicted by placing a source at an arbitrary seat location and applying the regression model to calculate relative exposure at all other seat locations, which were summed to obtain a total exposure for the cabin.

¶¶
https://www.sae.org/publications/technical-papers/content/2021-01-0036/

*** MS2 virus when collected in SKC BioSamplers (as in the current study) showed similar resistance to sampling and environmental stresses as does Newcastle disease virus but greater resistance than influenza A and MHV coronavirus. However, the relative exposure method of the current study might diminish the importance of sampling resistance. In addition, the airborne transport behavior of virus aerosols depends on the aerodynamic diameter of the evaporated droplet nuclei and not on biologic properties of individual viruses.

††† Including requirements set by the Federal Aviation Administration (https://www.faa.gov) and recommendations from the Environmental Protection Agency (https://www.epa.gov), and American Society of Heating, Refrigeration, and Air-Conditioning Engineers (https://www.ashrae.org). Additional information is available at https://www.faa.gov/data_research/research/med_humanfacs/cer/media/In-FlightOnboardMonitoring.pdf and https://www.nap.edu/catalog/10238/the-airliner-cabin-environment-and-the-health-of-passengers-and-crew/FlightOnboardMonitoring.pdf

§§§
https://www.epa.gov/indoor-air-quality-iaq/what-hepa-filter-1

¶¶¶ Applicability to COVID-19 transmission would involve two steps: MS2 phage exposure relating to SARS-CoV-2 exposure and SARS-CoV-2 exposure being large enough to cause COVID-19 transmission in a person. Relative exposure removes the importance of source specifics, making MS2 aerosol experiments a reasonable substitute in terms of transport physics. Importantly, the threshold behavior of COVID-19 transmission depends on factors related to in vivo respiratory droplets typically associated with SARS-CoV-2 and host characteristics such as individual susceptibility and is beyond the scope of this study.
